# Sodium channel current loss of function in induced pluripotent stem cell-derived cardiomyocytes from a Brugada syndrome patient

**DOI:** 10.1016/j.yjmcc.2017.10.002

**Published:** 2018-01

**Authors:** Elisabet Selga, Franziska Sendfeld, Rebecca Martinez-Moreno, Claire N. Medine, Olga Tura-Ceide, Sir Ian Wilmut, Guillermo J. Pérez, Fabiana S. Scornik, Ramon Brugada, Nicholas L. Mills

**Affiliations:** a Cardiovascular Genetics Centre, Department of Medical Sciences, University of Girona, Girona, Spain; b Institut d'Investigació Biomèdica de Girona (IDIBGI), Girona, Spain; c Centro de Investigación Biomédica en Red de Enfermedades Cardiovasculares (CIBERCV), Spain; d Scottish Centre for Regenerative Medicine, University of Edinburgh, United Kingdom; e BHF/University Centre for Cardiovascular Sciences, University of Edinburgh, United Kingdom; f Department of Pulmonary Medicine, Hospital Clinic-Institut d'Investigacions Biomèdiques August Pi i Sunyer (IDIBAPS), University of Barcelona, Barcelona, Spain; g Centro de Investigación Biomédica en Red de Enfermedades Respiratorias, University of Barcelona, Spain; h Hospital Josep Trueta, Girona, Spain

**Keywords:** Brugada syndrome, Pluripotent stem cells, Cardiomyocytes, Electrophysiology, Sodium current, AFP, alpha-fetoprotein, CPVT, catecholaminergic polymorphic ventricular tachycardia, cTnI, cardiac troponin I, cTnT, cardiac troponin T, EB, embryoid body, HEK, human embryonic kidney cells, iPS, induced pluripotent stem cells, iPS-CM, iPS cell-derived cardiomyocytes, LQT, long QT syndrome, *I*_Na_, sodium current, SNP, single nucleotide polymorphism, TEM, transmission electron microscopy, tsA201 cells, immortalized HEK293 cells

## Abstract

Brugada syndrome predisposes to sudden death due to disruption of normal cardiac ion channel function, yet our understanding of the underlying cellular mechanisms is incomplete. Commonly used heterologous expression models lack many characteristics of native cardiomyocytes and, in particular, the individual genetic background of a patient. Patient-specific induced pluripotent stem (iPS) cell-derived cardiomyocytes (iPS-CM) may uncover cellular phenotypical characteristics not observed in heterologous models. Our objective was to determine the properties of the sodium current in iPS-CM with a mutation in *SCN5A* associated with Brugada syndrome.

Dermal fibroblasts from a Brugada syndrome patient with a mutation in *SCN5A* (c.1100G > A, leading to Na_v_1.5_p.R367H) were reprogrammed to iPS cells. Clones were characterized and differentiated to form beating clusters and sheets. Patient and control iPS-CM were structurally indistinguishable. Sodium current properties of patient and control iPS-CM were compared. These results were contrasted with those obtained in tsA201 cells heterologously expressing sodium channels with the same mutation.

Patient-derived iPS-CM showed a 33.1–45.5% reduction in *I*_Na_ density, a shift in both activation and inactivation voltage-dependence curves, and faster recovery from inactivation. Co-expression of wild-type and mutant channels in tsA201 cells did not compromise channel trafficking to the membrane, but resulted in a reduction of 49.8% in sodium current density without affecting any other parameters.

Cardiomyocytes derived from iPS cells from a Brugada syndrome patient with a mutation in *SCN5A* recapitulate the loss of function of sodium channel current associated with this syndrome; including pro-arrhythmic changes in channel function not detected using conventional heterologous expression systems.

## Highlights

• iPS-CM were generated from a Brugada Syndrome patient who carries an SNV in SCN5A.
• Patient-specific iPS-CM show a loss of function of the sodium current.
• Use of iPS-CM uncovers changes in *I*_Na_ properties not apparent in tsA201 cells.

## 1 Introduction

Brugada syndrome is an autosomal dominant hereditary condition that is responsible for 20% of sudden cardiac deaths of patients with structurally normal hearts [1]. It is characterized by an abnormal electrocardiogram with ST-segment elevation in the right precordial leads V_1_ to V_3_ and right bundle-branch block frequently leading to ventricular fibrillation [2]. Patients often present symptoms of ventricular tachycardia, bradycardia, and atrial ventricular node conduction disorder, and more males than females are diagnosed with Brugada syndrome. To date, the implantation of a cardioverter defibrillator is the only proven effective treatment of the disease [3], [4].

Whilst Brugada syndrome has been associated with mutations in 23 genes [5], the majority of these disease-related mutations have been found in *SCN5A* [6]. This gene encodes the alpha-subunit of the cardiac sodium channel (Na_v_1.5) which is responsible for the sodium inward current (*I*_Na_). Heterologous expression of recombinant Na_v_1.5 channels in conventional cellular systems has provided invaluable insight into the molecular and electrophysiological basis of Brugada syndrome. Still, the main limitation of this approach is that the cells typically used (i.e., HEK293 cells, *Xenopus* oocytes) deviate considerably from human cardiomyocytes in many relevant aspects. These cells do not reflect the modulatory effects of accessory channel subunits or the influence of potential compensatory pathways, both of which could take place in native cardiomyocytes. Thus, studies of mutant channels using such expression systems might be missing important characteristics of native cardiomyocytes relevant to pathophysiology.

The differentiation of induced pluripotent stem (iPS) cells from patients with cardiac diseases into cardiomyocytes (iPS-CM) provides a cell model highly homologous to native human cardiomyocytes. The use of these surrogate cells allows investigators to study mutant ion channels in their native patient-specific cell environment. This includes all their regulatory proteins, and importantly, a physiologically controlled level of protein expression. To date, several cardiac channelopathies including long QT syndrome (LQT), catecholaminergic polymorphic ventricular tachycardia and Timothy syndrome have been modeled using the iPS cell approach [7]. Likewise, Davis et al. used iPS-CM to model an overlap LQT/Brugada syndrome [8]. Recently, BrS was modeled using patient-specific iPS-CM [9]. However, to date no reports exist that provide a complete characterization of the sodium current properties in Brugada syndrome patient-specific iPS-CM.

We generated iPS-CM from a patient diagnosed with Brugada Syndrome who carries a heterozygous missense mutation in *SCN5A* (c.1100G > A, leading to Na_v_1.5_p.R367H). This mutation had been previously found in Brugada Syndrome patients [10], [11]. Moreover, recombinant channels with this mutation were previously studied in homozygosis in both HEK293 cells and *Xenopus* oocytes [10], [11], [12], [13], [14]. These studies showed a total loss of function of the sodium current. Thus, we expected that, in iPS-CM, the presence of the Na_v_1.5_p.R367H mutation in heterozygosis would cause a decrease near 50% of the total current due to the expression of non-functional channels translated from the mutant allele. To assess this assumption, we analyzed and compared sodium current properties of iPS-CM derived from the patient and from a healthy individual without this mutation.

## 2 Materials and methods

Detailed experimental procedures are available in the Online Data Supplement.

### 2.1 Isolation and reprogramming of fibroblasts to induced pluripotent stem (iPS) cells

This study was approved by the South East Scotland Research Ethics Committee REC reference 11-SS-0095 and written informed consent was obtained from the two subjects included in the study. Dermal biopsies were dissected into 1 mm^3^ pieces, which were transferred to culture plastic, covered with a glass coverslip and cultured for 2 weeks before harvest. 5 × 10^5^ fibroblasts were reprogrammed with the Addgene episomal vectors pCXLE-hOCT3/4-shp53-F (encoding for Oct4 and shp53, Addgene plasmid # 27077), pCXLE-hSK (Sox2 and Klf, Addgene plasmid # 27078) and pCXLE-hUL (LMyc and Lin28, Addgene plasmid # 27080). Reprogrammed fibroblasts were replated onto 0.1% gelatin and medium was changed to stem cell selection medium (TeSR-E8, STEMCELL Technologies SARL, Grenoble, France) 7 days post electroporation. Colonies appeared 15–20 days later. Individual clones were picked into Matrigel (BD Biosciences, Franklin Lakes, NJ, USA) coated tissue culture plates and maintained in TeSR-E8.

### 2.2 Sequencing

For all iPS cell lines, the whole coding region of *SCN5A* was amplified (Verities PCR, Applied Biosystems, Austin, TX, USA), the PCR products were purified (ExoSAP-IT, Affymetrix, Inc. USB® Products, Cleveland, OH, USA) and they were directly sequenced in both directions (Big Dye Terminator v3.1 cycle sequencing kit and 3130XL Genetic Analyzer, both from Applied Biosystems). DNA sequences obtained were compared with *SCN5A* reference sequence NM_198056.2 using SeqScape v2.6 (Applied Biosystems).

### 2.3 Real-time quantitative reverse transcription PCR (qPCR)

RNA extraction and cDNA synthesis were carried out according to manufacturer's specifications (Cambio, Cambridge, UK and Applied Biosystems-Thermo Fisher Scientific). qPCR reactions were set up using the GoTaq qPCR Master Mix kit (Promega, Madison, WI, USA), and included a reference dye. The samples were analyzed in biological triplicates using the primers listed in Supplemental Table 2 and run in a Rotor-Gene 6000 (Corbett Life Science-Qiagen, Manchester, UK). Expression levels were determined by the ΔΔCt method.

### 2.4 Flow cytometry

Undifferentiated iPS cells were double stained with fluorochrome-conjugated antibodies against SSEA-3 (Alexa Fluor 488 conjugate, BioLegend, San Diego, CA, USA) and SSEA-4 (Phycoerythrin conjugate, BD Biosciences) for 30 min at room temperature. Samples were run on a FACS Fortessa Flow Cytometry System acquiring a minimum of 10,000 events. Data was analyzed using FlowJo software (Treestar, Inc., San Carlos, CA, USA).

### 2.5 Single nucleotide polymorphism (SNP) analysis

Genomic DNA was isolated from undifferentiated iPS cells using the MasterPure™ Complete DNA & RNA Purification Kit (Cambio). Single nucleotide polymorphism (SNP) analysis was performed by AROS Applied Biotechnology A/S (Aarhus, Denmark) using the Illumina CytoSNP-12 array. Data was visualized and analyzed using the GenomeStudio V2011.1 software.

### 2.6 Differentiation

Embryoid bodies (EBs) were formed from confluent undifferentiated iPS cell colonies. Collagenase treatment and mechanical disruption resulted in roughly evenly sized clumps that were cultured in suspension on non-treated tissue culture dishes in serum-supplemented medium for 7 days at 37 °C and 5% CO_2_. After a further 10 days on 0.1% gelatin coated tissue culture dishes, EBs were fixed and stained for the 3 germ layers.

For guided cardiac differentiation, two different protocols were used. First, a modified version of a published protocol [15] was implemented. In short, EBs were formed in supplemented Knock-out Dulbecco's Modified Eagle Medium, and cultured for 4 days in suspension and a further 10 days on 0.1% gelatin. Beating bodies started to emerge around day 8 after EB formation. Beating bodies were disaggregated using a previously published protocol [16], [17].

The second protocol used was monolayer-based [18]. Briefly, iPS cells were allowed to grow to 85–90% confluence. Then, culture media was changed to differentiation medium containing CHIR99021 (Day 0). Twenty-four hours later, media was changed to differentiation medium with heparin. From day 2 to day 5 the media contained both heparin and IWP2, and was renewed daily. At day 6, media was changed to differentiation medium with heparin only. The day after, the differentiation medium contained heparin and insulin. From day 8 onwards, cells were fed differentiation medium with insulin, and media was renewed every 2 days. iPS-CM started beating around day 8–10. iPS-CM monolayers were disaggregated with 0.25% trypsin-EDTA.

### 2.7 Immunocytochemistry

Undifferentiated cells were fixed in 4% paraformaldehyde for 10 min, permeabilized with 0.1% Triton X-100, blocked in 3% horse serum and stained for Nanog (R&D Systems, Abingdon, UK), Oct3/4 and Tra-1-60 (both from SantaCruz Biotechnology, Dallas, TX, USA). EBs differentiated to the three germ layers were fixed in cold methanol, permeabilized and blocked with 10% goat serum and stained for α-fetoprotein, β-tubulin III (both from Sigma-Aldrich, St. Louis, MO, USA) and Muscle actin (Dako, Glostrup, Denmark). Disaggregated beating bodies were fixed in 4% paraformaldehyde, permeabilized with 0.5% Triton X-100, blocked in 3% goat serum and stained for cardiac troponin T, cardiac troponin I, and alpha actinin (all from Abcam, Cambridge, UK). Appropriate secondary antibodies were used and cells were counterstained and mounted with ProLong Gold Antifade Reagent with DAPI.

### 2.8 Transmission electron microscopy (TEM)

Beating bodies were fixed in 2.5% glutaraldehyde in 4% PFA, washed with 0.1 mol/L Phosphate buffer, post-fixed in 1% Osmium Tetroxide in 0.1 mol/L Phosphate buffer, then washed with 0.1 mol/L Phosphate buffer. Samples were dehydrated in 50%, 70%, 90% and 100% ethanol, then in propylene oxide, and embedded in Araldite resin. Sections, 1 μm thick, were cut on a Reichert OMU4 ultramicrotome, stained with Toluidine Blue, and viewed in a light microscope to select suitable areas for investigation. Ultrathin sections, 60 nm thick, were cut from selected areas, stained in Uranyl Acetate and Lead Citrate then viewed in a Philips CM120 Transmission electron microscope. Images were taken on a Gatan Orius CCD camera.

### 2.9 Sodium current recordings in iPS-CM

Beating bodies and monolayers were disaggregated and plated onto 1% gelatin. Whole cell sodium currents were measured at room temperature either using the perforated patch-clamp technique 24–48 h after disaggregation (for beating bodies) or using the whole cell technique 24-96 h after disaggregation (for monolayers). Voltage clamp experiments were controlled and analyzed with an Axopatch 200B amplifier and pClamp 10.2/Digidata 1440A acquisition system (Molecular Devices, Sunnyvale, CA, USA) and OriginPro8 software (OriginLab Corporation, Northampton, MA, USA). Data were filtered at 5 kHz and sampled at 5–20 kHz. Activation curve data were fitted to a Boltzmann equation, of the form *g* = *g*_max_ / (1 + exp(*V*_1/2_ − *V*_m_) / *k*), where *g* is the conductance, *g*_max_ the maximum conductance, *V*_m_ is the membrane potential, *V*_1/2_ is the voltage at which half of the channels are activated and *k* is the slope factor. Steady-state inactivation values were fitted to a Boltzmann equation of the form *I* = *I*_max_/(1 + exp(*V*_1/2_ − *V*_m_)/*k*), where *I* is the peak current amplitude, *I*_max_ the maximum peak current amplitude, *V*_m_ is the membrane potential, *V*_1/2_ is the voltage at which half of the channels are inactivated, and *k* is the slope factor. The sodium current decay after the peak *I*_Na_ was fitted with a monoexponential function between − 40 and − 25 mV, and a bi-exponential function between − 20 and 20 mV, from where τ fast and τ slow were obtained. Both the slow inactivation and the recovery from inactivation data were fitted to mono-exponential functions, to obtain their respective time constants.

### 2.10 Sodium current recordings in tsA201 cells

#### 2.10.1 Site-directed mutagenesis

*SCN5A*_c.1100G > A was introduced in the wild-type (WT) human *SCN5A* cDNA cloned in pcDNA3.1 using the QuikChange Site-Directed Mutagenesis system (Stratagene, La Jolla, CA, USA). The resultant construct was directly sequenced to verify the presence of the desired mutation and the absence of additional variations.

#### 2.10.2 Cell culture and transfection

tsA201 cells were maintained as described previously [19] and transiently transfected with the vectors encoding for *SCN5A* (Na_v_1.5_WT only, Na_v_1.5_R367H only, or both WT and R367H vectors), using Lipofectamine 2000 (Life Technologies).

#### 2.10.3 Electrophysiological studies

Sodium currents were measured at room temperature using the standard whole cell patch-clamp technique [20] 48 h after transfection. Voltage clamp experiments were controlled and analyzed as described for iPS-CM.

### 2.11 Cell surface protein biotinylation in tsA201 cells

Cells were plated and transfected as above and membrane proteins were biotinylated and isolated as previously described [19]. Proteins were resolved in 4% SDS-PAGE gels and transferred to PVDF membranes. Membranes were probed with an anti-human Na_v_1.5 antibody (Alomone Labs, Jerusalem, Israel) and a secondary horseradish peroxidase-conjugated antibody (Thermo Scientific, Rockford, IL, USA). Signals were detected with the SuperSignal West Femto Chemiluminiscent substrate (Pierce, Thermo Scientific). A mouse antibody against Na^+^/K^+^ ATPase (Abcam) was used as biotinylation control. Expression of Na_v_1.5 was quantified using ImageJ software (National Institute of Health, NIH).

### 2.12 Statistical analysis

Results are presented as mean ± standard error. Statistical comparisons were performed using an unpaired Student's *t*-test or two-way ANOVA with Bonferroni or Dunnett's post-test for multiple comparisons as appropriate. Statistical significance was defined where *p* < 0.05.

## 3 Results

### 3.1 Generation of iPS cell lines

**Fig. 1:**
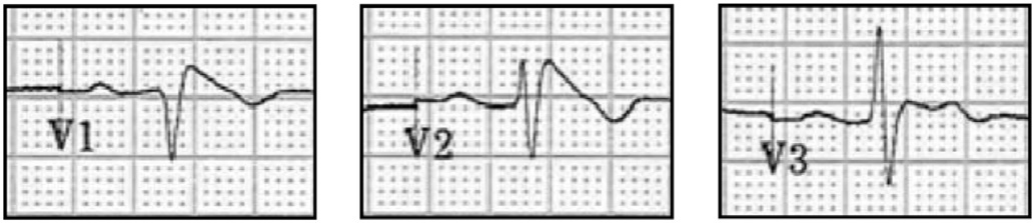
Clinical manifestation of Brugada syndrome. Partial electrocardiogram of the proband showing the ST segment elevation characteristic of Brugada syndrome.

**Fig. 2:**
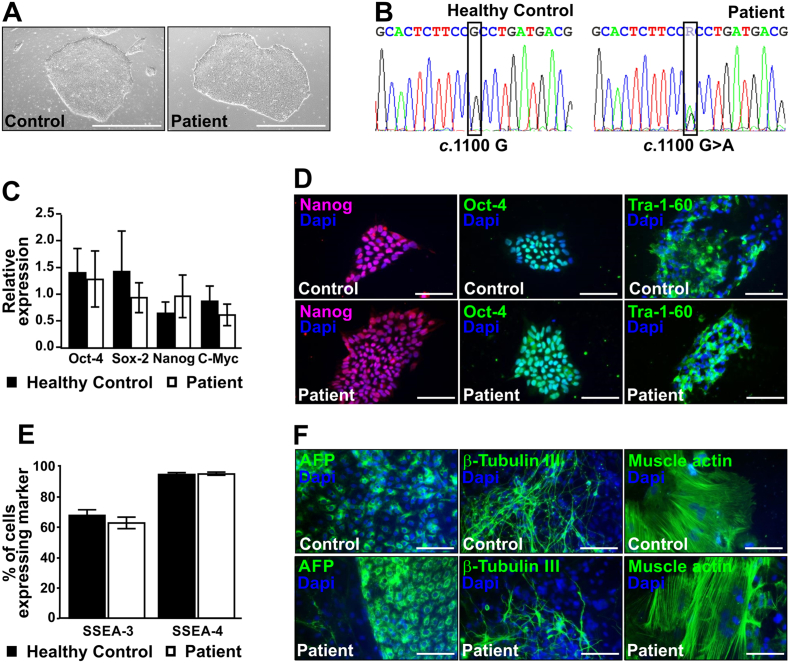
Control and patient-specific iPS cell lines express pluripotency markers. (A) Representative light microscopy images of healthy control (left) and patient (right) newly derived induced pluripotent stem (iPS) cells in feeder-free culture. Scale bars represent 1000 μm. (B) The missense mutation *SCN5A*_*c*.1100G > A was identified in genomic DNA isolated from patient (right) but not healthy control derived iPS cells (left). (C) The relative expression of pluripotency transcription factors Oct-4, Sox2, Nanog and c-Myc in undifferentiated cells is shown as mean values of biological triplicates ± SEM. (D) Representative merged images of undifferentiated iPS cells showing positive staining for pluripotency markers Nanog, Oct-4 and Tra-1-60 for both control (upper panels) and patient (lower panels) iPS cells. Nuclei were counterstained with DAPI (blue). Scale bars represent 100 μm. (E) The percentage of SSEA-3 and SSEA-4 positive cells in undifferentiated iPS cell cultures is given as the mean of at least three independent wells ± SEM (F) Embryoid bodies (EBs) were formed from undifferentiated iPS cells and differentiated in suspension for 7 days and for an additional 10 days adhered to plates. Control (upper panels) and patient (lower panels) derived EBs showed positive staining for all three germ layers endoderm (AFP), ectoderm (β-tubulin III) and mesoderm (muscle actin). Nuclei were counterstained with DAPI (blue). Scale bars represent 100 μm.

A male Caucasian patient, aged 69, clinically diagnosed with Brugada syndrome was identified through the Familial Arrhythmia Network for Scotland. He presented with classical ST-segment elevation in the electrocardiogram (Fig. 1) and was found to have a missense mutation in *SCN5A* (c.1100G > A, leading to Na_v_1.5_p.R367H) in heterozygosis. This mutation is located in the pore region of the first domain of Na_v_1.5. We obtained and processed in parallel skin biopsies from this patient and an age and sex-matched healthy volunteer. Emerging fibroblasts were reprogrammed using episomal vectors and, under stem cell selection conditions, possible iPS cell colonies, assessed based on morphology, appeared after around 20 days. Colonies were treated as individual clones, manually picked and expanded in feeder-free conditions to give rise to independent cell lines. Each cell line grew in flat densely packed colonies with cells exhibiting the high nucleus to cytoplasm ratio typical for embryonic stem cells (Fig. 2A). Sequencing of the whole coding region of *SCN5A* in genomic DNA samples of all cell lines confirmed the presence of *SCN5A*_c.1100G > A in the patient lines, and its absence in the control lines (Fig. 2B). We also identified 3 synonymous SNVs (rs6599230, rs7430407 and rs1805126) that were present in all lines.

### 3.2 Characterization of patient and control iPS cell lines

To verify that the iPS cell lines expressed pluripotency markers, RNA was extracted from undifferentiated cultures, purified and reverse transcribed for each sample in biological triplicate and assessed for the relative expression of *POU5F1* (the gene encoding Oct-4), *Sox2*, *Nanog* and *c-Myc* (Fig. 2C). One-way ANOVA showed no differences in expression of the tested pluripotency transcription factors between patient and control iPS cell lines. We also fixed and stained undifferentiated pluripotent stem cells for the pluripotency markers Nanog, Oct-4 and Tra-1-60. As expected, both patient and control cell lines showed nuclear Nanog and Oct-4 staining, and cell surface staining for Tra-1-60 (Fig. 2D). Differentiated cells surrounding the colonies did not stain for pluripotency markers but were visible as DAPI stained nuclei. Using flow cytometry, we also investigated the percentage of cells expressing the cell surface markers SSEA-3 and SSEA-4 in undifferentiated populations of patient and control iPS cell lines. No differences were observed in the percentage of cells expressing each marker between patient and control cell lines (Fig. 2E). Additionally, we analyzed genomic DNA from each iPS cell line for chromosomal integrity using a single nucleotide polymorphism (SNP) array (Supplemental Table 1). Finally, we generated embryoid bodies (EBs) from both patient and control derived iPS cell lines and assessed their ability to differentiate into all 3 germ layers. EBs spontaneously differentiated into cells from endoderm, ectoderm and mesoderm, as evidenced by immunofluorescence staining for alpha-fetoprotein (AFP), β-tubulin III and muscle actin (Fig. 2F).

### 3.3 Morphological characterization of patient and control derived cardiomyocytes

**Fig. 3:**
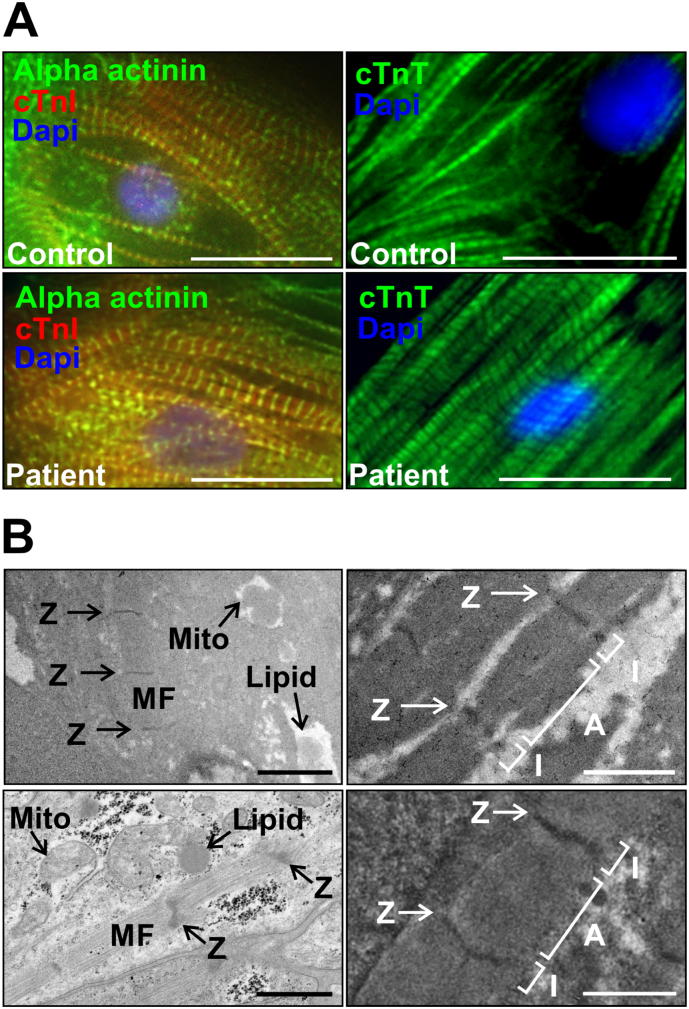
Control and patient-specific iPS-CM show typical patterns for cardiac markers. (A) Control (top panels) and patient (bottom panels) iPS-CM stained positive for alpha actinin and cardiac Troponin I (left), and cardiac Troponin T (right), showing intact myofibrils. Nuclei were counterstained with DAPI (blue). Figure shows representative merged images. Scale bars represent 20 μm. (B) Control (top panels) and patient (bottom panels) iPS-CM showed mitochondria (Mito) and lipid deposits (Lipid) as well as myofibrils (MF) of varying width and length (left). We also observed sarcomeres with I bands (I) and A bands (A) in addition to Z lines (Z) (right). Scale bars represent 1 μm.

We induced cardiac differentiation of iPS cells and obtained cardiomyocytes (iPS-CM) which were aggregated in the form of beating bodies, and contracted spontaneously (Supplementary Video 1, Supplementary Video 2). To compare the morphological properties of patient and control iPS-CM, we enzymatically and mechanically disaggregated 3–4 week old beating bodies for immunofluorescence staining. Disaggregation resulted in single as well as clusters of beating cardiomyocytes, with groups of connected cardiomyocytes exhibiting synchronized contraction. After 3–5 days of recovery, we fixed and stained cells for markers of the cardiac contractile apparatus (Fig. 3A). Control and patient cells isolated from beating bodies stained positive for alpha actinin, cardiac troponin I (cTnI) and cardiac troponin T (cTnT). Single cTnT staining exhibited the striated pattern of thin filaments while double staining for cTnI and alpha actinin resulted in alternating striations, staining thin filaments and *Z*-lines respectively.

We examined the structure of the contractile apparatus of beating bodies in more detail using transmission electron microscopy. In both patient and control iPS-CM, myofibrils varied in size with some consisting of a single sarcomere. Some cells contained a greater number of myofibrils with increased organization, exhibiting some alignment of *Z*-lines between adjacent myofibrils (Fig. 3B, left). At high magnification, we observed sarcomeres with I-bands and A-bands in addition to Z-lines (Fig. 3B, right).

### 3.4 Electrophysiological characterization of patient and control derived cardiomyocytes

**Fig. 4:**
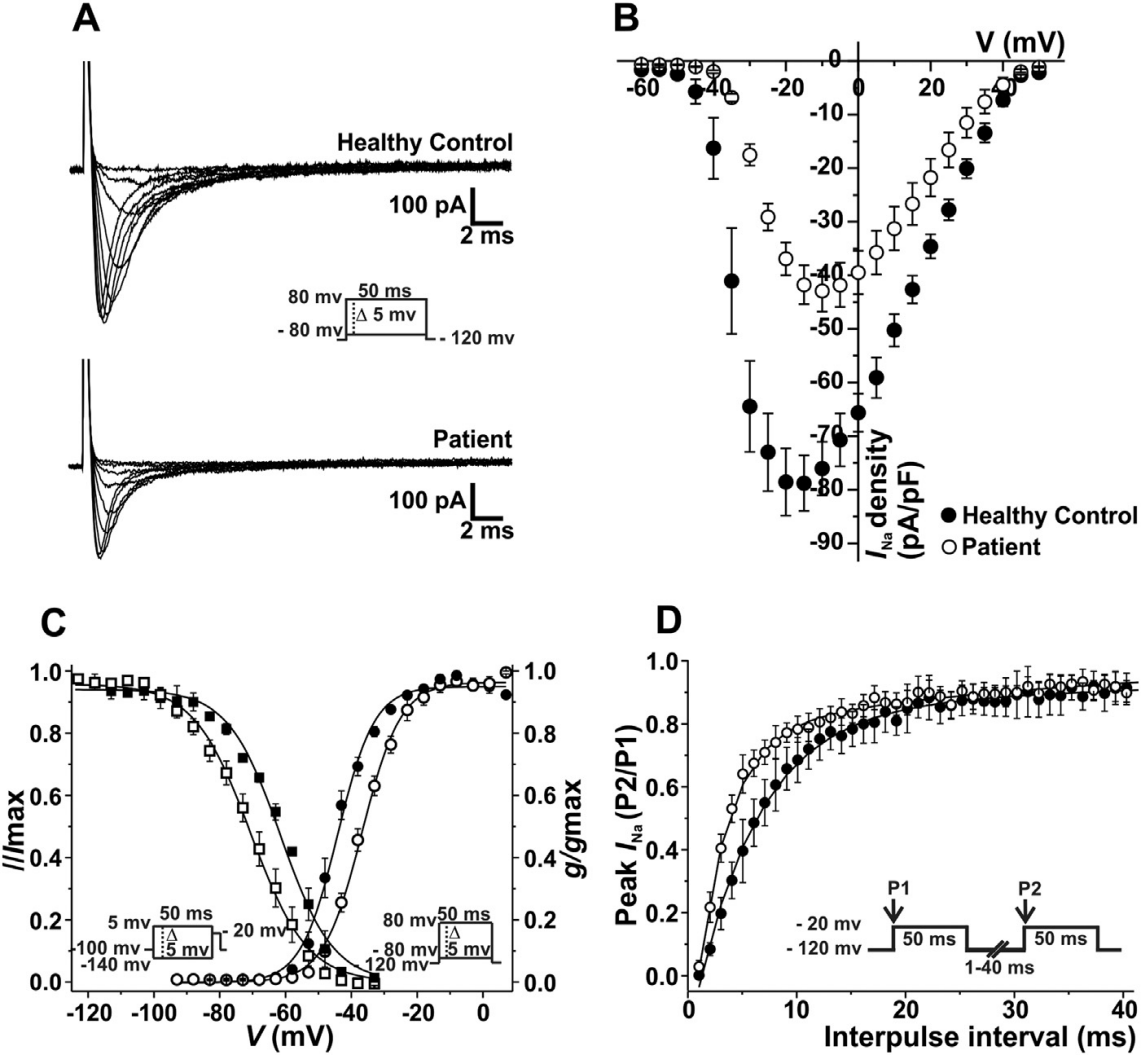
Patient-specific iPS-CM display altered *I*_Na_ properties. Filled symbols are used to depict data for healthy control iPS-CM and open symbols represent values for patient-specific cardiomyocytes. Values are expressed as mean ± SEM. (A) Representative whole cell sodium current traces recorded from control and patient-specific cells. Currents were elicited by depolarizing potentials as shown in the inset. Traces for pulses from − 50 mV to − 15 mV are shown. (B) Current-voltage (I–V) relationship. *I*_Na_ amplitude was normalized to the cell capacitance to obtain current density (*I*_Na_ density) values. Experimental points represent the peak-amplitude of *I*_Na_ density at each given voltage. (C) *I*_Na_ voltage-dependence of activation and steady-state inactivation for control and patient cells. Conductance values for the activation curve were obtained from the peak current values taken from the I–V relationship. Symbols represent experimental data plotted against the given depolarizing voltage values. Steady-state inactivation protocol is shown in the inset on the left. Symbols represent experimental data plotted against preconditioning pulse values. Solid lines represent the Boltzmann fit of the experimental points. (D) Recovery from inactivation properties were studied by applying the double pulse protocol shown in the inset. A 50 ms depolarizing pulse to − 20 mV (P1) was followed by a hyperpolarizing pulse to − 120 mV of increasing duration (1–40 ms), that preceded a test pulse to − 20 mV (P2). The P2/P1 ratio values plotted against the recovery interval times were fitted to bi-exponential functions (solid lines).

We first studied the sodium current properties of patient and control iPS-CM generated using an embryoid body based differentiation protocol. Cardiomyocytes were derived from two different patient iPS cell clones. Two separate differentiations for each clone were used for our study. All these iPS-CM were characterized independently. The sodium current properties of each of the lines were compared to the results obtained from cardiomyocytes derived from one control iPS cell line. Since the results obtained for the two differentiations per clone and for the two patient iPS-CM clones were very similar, data was pooled. Whole cell current recordings showed a loss of function of the sodium channel current in patient-derived compared to control-derived cardiomyocytes. Representative examples of *I*_Na_ traces for patient and control cardiomyocytes at varying potentials are shown in Fig. 4A. Peak *I*_Na_ density was reduced by 45.5% in patient-derived compared to control-derived cardiomyocytes (p < 0.05, Fig. 4B, Table 1). In addition, voltage-activation data fit to a Boltzmann equation revealed a positive 7.42 mV shift of the *V*_1/2_ in patient respect to control cells, without significant changes in the slope factor (Fig. 4C, Table 1). Steady-state voltage dependence of inactivation data fit to a Boltzmann equation evidenced a negative shift of 8.51 mV of the *V*_1/2_ in patient respect to control cells, and no significant changes in the slope factor (Fig. 4C, Table 1). Analysis of recovery from inactivation time course is illustrated in Fig. 4D. Data was fitted with a bi-exponential function and revealed a faster recovery in the patient cells (Table 1).

**Table 1:** Biophysical parameters of WT and mutant channels in iPS-CM.

Parameter	Beating bodies		Monolayer	
	Healthy control iPS-CM	Patient iPS-CM	Healthy control iPS-CM	Patient iPS-CM
Peak *I*_Na_				
pA/pF	− 78.77 ± 5.16	− 42.93 ± 3.86⁎⁎⁎	− 45.62 ± 5.37	− 30.51 ± 3.09⁎
*n*	3	7	11	13
Activation				
*V*_1/2_ (mV)	− 44.15 ± 0.37	− 36.73 ± 0.32⁎⁎⁎	− 32.96 ± 0.79	− 25.44 ± 0.78⁎⁎⁎
*k*	5.57 ± 0.33	5.86 ± 0.28	3.94 ± 0.14	5.58 ± 0.26⁎⁎⁎
*n*	3	7	11	13
Steady-state inactivation				
*V*_1/2_ (mV)	− 61.64 ± 0.77	− 70.15 ± 2.76⁎	− 48.80 ± 0.79	− 54.69 ± 1.21⁎⁎
*k*	8.94 ± 1.54	8.70 ± 0.57	6.70 ± 0.33	9.59 ± 0.24⁎⁎⁎
*n*	3	7	8	10
Recovery from inactivation				
*τ* (ms)	τf: 5.85 ± 1.01 τs: 40.38 ± 4.95	τf: 2.89 ± 0.35⁎⁎ τs: 34.45 ± 14.45	τf: 2.58 ± 0.31 τs: 46.17 ± 7.01	τf: 1.68 ± 0.18⁎ τs: 20.12 ± 6.76⁎
*n*	3	6	5	5

Activation and steady-state inactivation parameters were calculated by data fitting to Boltzmann functions (see Materials and methods). *V*_1/2_ is the voltage for half-maximal activation or steady-state inactivation, *k* is the slope factor and *n* the number of cells. Recovery from inactivation data was fitted to a bi-exponential function (see Materials and methods) to obtain the fast and slow time constants (*τ*f and *τ*s, respectively). Values are expressed as mean ± SEM.

⁎ p < 0.05.

⁎⁎ p < 0.01.

⁎⁎⁎ p < 0.001.

In a second set of experiments, we studied the sodium current properties of patient and control iPS-CM generated using a monolayer-based differentiation protocol. For these experiments, we used one of the patient iPS cell lines and an additional healthy control iPS cell line (Supplementary Video 3, Supplementary Video 4). Whole cell current recordings showed a loss of function of the sodium channel current in patient-derived compared to control-derived cardiomyocytes. Examples of *I*_Na_ traces are depicted in Supplemental Fig. 1A. Analysis of the data revealed a reduction of 33.12% in peak current density in the patient-derived compared to control-derived iPS-CM (p < 0.05; Supplemental Fig. 1B and Table 1). It is worth noting that this analysis shows an underestimation of the current reduction since 6 out of 17 control cells displayed currents too large to allow proper voltage control in our already low sodium recording conditions. Furthermore, we observed a positive 7.53 mV shift in the activation *V*_1/2_, and a negative 5.89 mV shift in the steady-state inactivation *V*_1/2_ in patient respect to control cells (p < 0.001 and p < 0.01, respectively; Supplemental Fig. 1C and Table 1). As in the first set of experiments, we observed a faster recovery from inactivation in the patient iPS-CM respect to control (p < 0.05, Supplemental Fig. 1D and Table 1).

### 3.5 Electrophysiology and cell surface protein biotinylation in tsA201 cells

It is worth noting that the available studies of Na_v_1.5_p.R367H had been performed in a homozygous state for either the WT or the mutant gene (WT/WT or R367H/R367H). They showed a total loss of *I*_Na_ when only mutant channels were expressed, both in HEK293 cells and in *Xenopus* oocytes. Conversely, our patient carried the mutant channel in a heterozygous state (WT/R367H), and our results in iPS-CM showed differences in the biophysical properties of the current obtained in the patient compared to the control cells. This prompted us to study the biophysical properties of the sodium current in tsA201 cells transiently expressing sodium channels in heterozygous conditions. In contrast with the iPS-CM, in this setting the only difference between cells would be the point mutation of interest.

**Fig. 5:**
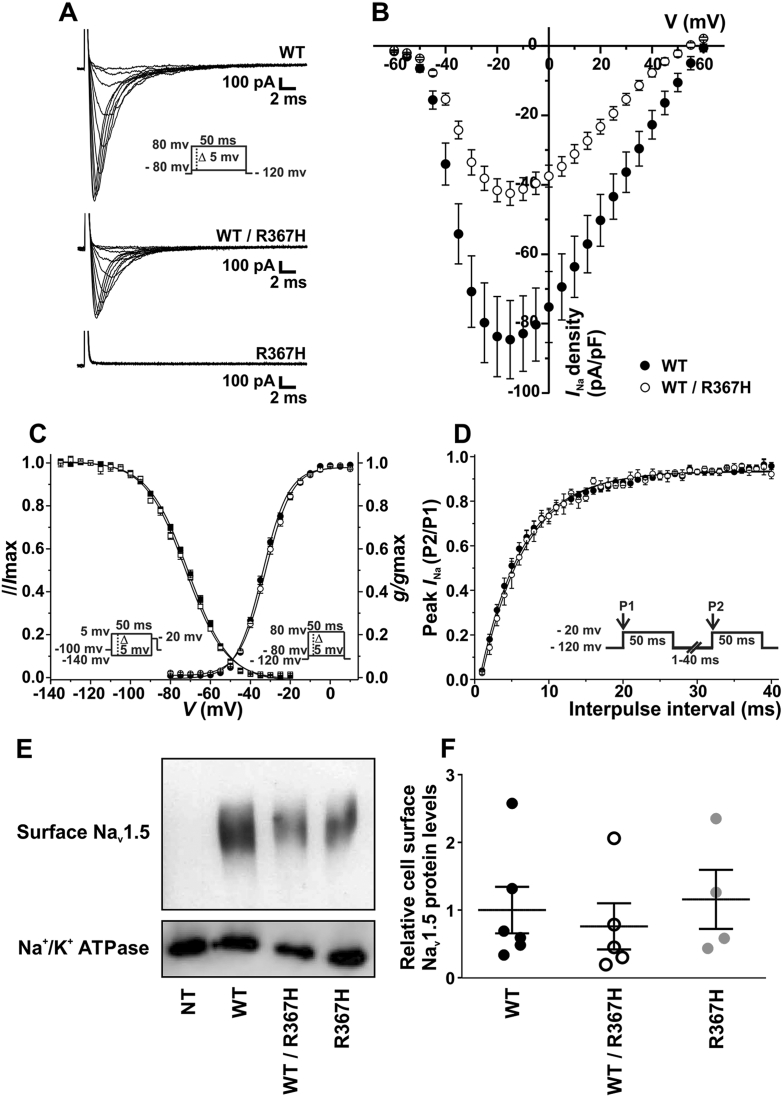
Na_v_1.5_R367H markedly decreases peak *I*_Na_. (A) Representative whole cell sodium current traces recorded from WT, WT/R367H and R367H cells. Currents were elicited by depolarizing potentials as shown in the inset. Traces for pulses from − 60 mV to − 5 mV are shown. (B–D) Filled symbols are used to depict data for WT cells, and open symbols represent values for WT/R367H cells. Values are expressed as mean ± SEM. (B) Current-voltage (I–V) relationship. *I*_Na_ amplitude was normalized to the cell capacitance to obtain current density (*I*_Na_ density) values. Experimental points represent the peak-amplitude of *I*_Na_ density at each given voltage. (C) *I*_Na_ voltage-dependence of activation and steady-state inactivation for WT and WT/R367H cells. Conductance values for the activation curve were obtained from the peak current values taken from the I–V relationship. Symbols represent experimental data plotted against the given depolarizing voltage values. Steady-state inactivation protocol is shown in the inset on the left. Symbols represent experimental data plotted against preconditioning pulse values. Solid lines represent the Boltzmann fit of the experimental points. (D) Recovery from inactivation properties were studied by applying the double pulse protocol shown in the inset. A 50 ms depolarizing pulse to − 20 mV (P1) was followed by a hyperpolarizing pulse to − 120 mV of increasing duration (1–40 ms), that preceded a test pulse to − 20 mV (P2). The P2/P1 ratio values plotted against the recovery interval times were fitted to mono-exponential functions (solid lines). (E) Representative image of western blot detection of Na_v_1.5 and Na^+^/K^+^ ATPase proteins performed after cell surface biotinylation of non-transfected cells (NT), cells transfected only with the vector encoding Na_v_1.5_WT (WT), with the vectors encoding Na_v_1.5_WT and Na_v_1.5_R367H (WT/R367H) and only with the vector encoding Na_v_1.5_R367H (R367H). (F) Scatter plot showing the relative surface Na_v_1.5 protein expression. Intensity values were calculated as described in Methods and normalized relative protein expression was plotted for each of the replicates (dots). Lines represent means ± SEM.

We transfected tsA201 cells with either the vector encoding Na_v_1.5_WT (homozygous WT cells), the vector encoding Na_v_1.5_R367H (homozygous R367H cells) or both vectors (heterozygous WT/R367H cells). We performed the electrophysiological recordings on isolated cells 48 h after transfection using whole cell patch-clamping and normal sodium bath solution. Representative examples of *I*_Na_ traces for the 3 conditions are shown in Fig. 5A. Recordings performed in cells transfected with only Na_v_1.5_R367H revealed a complete loss of current (Fig. 5A, lower panel). Peak *I*_Na_ density was significantly reduced by 49.8% in heterozygous WT/R367H cells with respect to homozygous WT/WT cells (Fig. 5B). No further changes in *V_1/2_* were observed after data fitting in either activation or steady-state inactivation (Fig. 5C, Table 2) or in the recovery from inactivation time course (Fig. 5D, Table 2).

**Table 2:** Biophysical parameters of WT and mutant channels in tsA201 cells.

Parameter	WT tsA201 cells	WT/R367H tsA201 cells
Peak *I_Na_*		
pA/pF	− 84.62 ± 11.24	− 42.48 ± 3.46⁎⁎
*n*	11	10
Activation		
*V*_1/2_ (mV)	− 33.91 ± 0.20	− 32.42 ± 0.22
*k*	6.54 ± 0.18	6.63 ± 0.20
*n*	11	10
Steady-state inactivation		
*V*_1/2_ (mV)	− 71.03 ± 0.25	− 72.36 ± 0.32
*k*	10.17 ± 0.23	10.18 ± 0.29
*n*	11	6
Recovery from inactivation		
*τ* (ms)	5.80 ± 0.17	5.93 ± 0.23
*n*	6	4

Activation and steady-state inactivation parameters were calculated by data fitting to Boltzmann functions (see Materials and methods). *V*_1/2_ is the voltage for half-maximal activation or steady-state inactivation, *k* is the slope factor and *n* the number of cells. Recovery from inactivation data was fitted to a mono-exponential function to obtain the time constant *τ*. Values are expressed as mean ± SEM.

⁎⁎ p < 0.01.

Previous immunofluorescence assays of Na_v_1.5_p.R367H expressed in HEK293 cells had shown that the mutant channels could reach the plasma membrane [14]. To quantify whether the WT and mutant channels localized at the cell surface in similar amounts, we performed protein biotinylation assays in tsA201 cells. Figs 5E and F show that the cell surface Na_v_1.5 expression was similar in homozygous Na_v_1.5_WT cells, homozygous Na_v_1.5_R367H cells and heterozygous WT/R367H cells. Therefore, the p.R367H Na_v_1.5 channels could traffic efficiently to the plasma membrane, and did not interfere in WT channel trafficking.

## 4 Discussion

Sodium channel current loss of function has been long and widely accepted as a hallmark of Brugada syndrome. Still, most of the evidence comes from heterologous expression studies. To date, only two studies have shown evidence for the association of a sodium channel mutation with either Brugada syndrome or overlapped LQT3/Brugada syndrome in iPS-CM [8], [9]. However, these studies only show a reduction in sodium current density in patient iPS-CM compared to the control iPS-CM. Thus, our present study is the first to show the loss of sodium channel function by a complete characterization of the sodium current properties in native cells from a BrS patient who carries a mutation in *SCN5A*. Although expected, it is not trivial to show in native cells that a mutation that causes total loss of function of the current provokes a reduction of the total current to 50%. In fact, we identified in the iPS-CM further pro-arrhythmic changes in channel function that could not be detected using conventional heterologous expression systems. It is to note that a comparison of the effects of *SCN5A* mutations on sodium current properties in both model systems had never been performed before for Brugada syndrome.

We successfully generated iPS cells through reprogramming of dermal fibroblasts from a Brugada syndrome patient and an age and sex-matched healthy volunteer. No differences were observed between patient and control iPS cell lines in terms of colony morphology, expression of pluripotency transcription factors and proteins, or their ability to differentiate into the three germ layers. As anticipated, the only difference was the presence of the patient's mutation (*SCN5A*_c.1100G > A) in the patient-specific iPS cell lines and its absence in those obtained from the healthy volunteer.

Comparison of ultrastructure and staining for markers of the cardiac contractile apparatus did not show any obvious differences between cardiomyocytes derived from patient-specific and healthy control pluripotent stem cells. Importantly, the analysis of electrophysiological recordings revealed a 33.1–45.5% reduction of peak *I*_Na_ density in patient-derived compared to healthy control-derived cardiomyocytes. This decrease represents a loss of function of the sodium channel. Previous findings had shown a complete loss of function of homozygous Na_v_1.5_R367H channels [10], [11], [12], [13]. In heterozygous conditions, a reduction of *I*_Na_ to 50%, most likely originated only from the expression of the WT allele, would be expected. This is consistent with reduced peak *I*_Na_ density that we observed in the patient iPS-CM. Surprisingly, our iPS-CM model showed additional changes in other electrophysiological properties. We observed a positive shift in the voltage-dependence of activation and a negative shift in the voltage-dependence of steady-state inactivation in the patient-specific cardiomyocytes. These voltage-dependence changes further contribute to the loss of function of the sodium channel. We also detected a faster recovery from channel inactivation in the patient iPS-CM. This acceleration in channel recovery has been proposed to contribute to the generation of arrhythmias in Brugada syndrome patients [21]. Importantly, we observed similar changes in sodium current properties when comparing patient and control iPS-CM generated with two distinct differentiation protocols. Although the differences in the current between patient and control iPS-CM were qualitatively the same in both experimental sets, the magnitude of the effects was different. This variability is expected considering that iPS-CM were obtained by different protocols. Moreover, the control iPS-CM used in both protocols were from two non-related healthy individuals. The results obtained support the idea that I) the SNV under study has an effect on sodium current properties; and II) the individual-specific genetic background could modulate the magnitude of this effect.

In support to our experimental data, we provide a comparison of the *I*_Na_ parameters reported here with those previously published for embryonic stem cell-derived cardiomyocytes (ES-CM), iPS-CM and native cardiomyocytes (Table 3). It is worth noting that the voltage for *I*_Na_ half-maximal activation that we recorded in our control cells is similar to that reported for native human cardiomyocyte preparations [22], [23], [24], [25], [26], [27] (Table 3). Our value for EB-differentiated cardiomyocytes was even closer to that for native cells than that of other human iPS [28], [29], [30] and ES [31], [32] cell derived cardiomyocytes. Regarding half-maximal steady-state inactivation voltage, the value reported for native preparations is clearly more hyperpolarized (by approx. 19 mV) than the one recorded in ES-CM, and this difference increases up to 33 mV when compared with that for iPS-CM. However, the values obtained in this study are in line with those reported for other iPS-CM (Table 3).

**Table 3:** Comparison of *I*_Na_ voltage-dependent properties.

	*V*_1/2_ activation (mV)	*V*_1/2_ steady-state inactivation(mV)
This study		
EB-based differentiation	− 44.15 ± 0.37 (*n* = 3)	− 61.48 ± 0.5 (*n* = 3)
Monolayer-based differentiation	− 32.96 ± 0.79 (*n* = 11)	− 48.80 ± 0.79 (*n* = 8)
iPS-CM		
Ma et al. [28]	− 34.1 (*n* = 5)	− 72.1 (*n* = 5)
Ma et al. [29]	− 39.68 ± 1.96 (*n* = 8)	− 44.63 ± 5.77 (*n* = 8)
Terrenoire et al. [30]	− 25 ± 0.3 (*n* = 3)	− 70.3 ± 1.7 (*n* = 3)
Terrenoire et al. [30]		− 68.9 ± 0.9 (*n* = 6)
ES-CM		
Satin et al. [31]	− 30 (*n* = 21)	− 72.6 ± 0.7 (*n* = 19)
Jonsson et al. [32]	− 34 (*n* = 7)	− 78 (*n* = 7)
Native cardiomyocytes		
Sakakibara et al. [23]	− 38.9 ± 0.9 (*n* = 46)	− 95.8 ± 0.9 (*n* = 46)
Sakakibara et al. [27]	− 42.3 ± 1.7 (*n* = 12)	− 99.8 ± 2.1 (*n* = 12)
Sakakibara et al. [27]	− 43.8 ± 0.2 (*n* = 10)	− 94.5 ± 2.3 (*n* = 10)
Feng et al. [26]	− 38.6 ± 2.9 (*n* = 6)	− 95.1 ± 5.4 (*n* = 6)
Valdivia et al. [24]	− 51 ± 1.0 (*n* = 11)	− 102 ± 16 (*n* = 3)
Valdivia et al. [24]	− 50 ± 1.1 (*n* = 17)	− 88 ± 1.9 (*n* = 10)
Barajas-Martínez et al. [25]	~− 52	− 90.1 ± 0.9 (*n* = 3)
Jia et al. [22]		− 93 (*n* = 5)

The table shows the voltage for half-maximal activation and inactivation for control cells in each category. Values are expressed as mean ± SD or SEM, as reported in each original study (if available). The number of cells characterized in each work is provided (*n*).

Our cell surface protein biotinylation studies in tsA201 cells demonstrate that both WT and mutant channels reach the cell surface to a similar extent when Na_v_1.5_R367H is expressed either in heterozygosis or homozygosis. Thus, the corresponding partial or total *I*_Na_ reduction would result from a mutant non-functional channel rather than by a trafficking defect, in agreement with previous studies [14]. Due to the limited amount of tissue available from iPS-CM, we could not perform biotinylation studies with these cells. However, based on our results from tsA201 cells, a trafficking defect in iPS-CM caused by the mutation would be unlikely. Consequently, these results argue against a negative dominant effect of p.R367H as it has been shown for other mutations in Na_v_1.5 [33], [34].

Nevertheless, a functional interaction between sodium channel alpha subunits could account for the differences in voltage-dependent and kinetic properties of the sodium current that we observed between patient and control iPS-CM. Indeed, recent structural studies of the Na_v_1.5 channel have shown evidence for the formation of asymmetric α-subunit dimers [35]. The authors propose that the dimerization of the α subunits would be mediated through the binding of calmodulin to their C-terminus. The mutation studied in the present study (p.R367H) is far from the C-terminus of the sodium channel, and thus would not be expected to interfere with dimerization. However, if as proposed, functional Na_v_ channels do exist in oligomeric states involving at least two α subunits, functional interactions between WT and mutant α subunits could explain the kinetic and voltage dependent differences in patient-specific iPS-CM compared to control. We did not observe such differences in current properties in tsA201 cells. This might be due to unbalanced amounts of endogenous calmodulin or other sodium channel modulatory molecules, and the overexpressed Na_v_1.5 in tsA201 cells. Conversely, iPS-CM are likely to possess the molecular machinery to allow Na_v_1.5 oligomerization. Thus, the formation of heterodimers (WT/R367H) could account for the differences in *I*_Na_ observed between the patient and control iPS-CM.

Overall, the two cellular models studied in this work present important differences. It should be considered that the experimental conditions for tsA201 cells (homozygous WT/WT vs. heterozygous WT/mutant) only differ in the transfected gene. On the other hand, the patient and the control iPS-CM are likely to carry variations in their genetic background in addition to the patient's mutation. Some of these variations could affect cardiac-specific proteins (e.g. channel auxiliary subunits) which are not expressed in tsA201 cells. These variations, either common or rare, could influence the sodium current's biophysical properties. Altogether, our work points to these differences as a possible explanation of why electrophysiological changes other than reduced peak *I*_Na_ were evident only when analyzing iPS-CM but not in the heterologous tsA201 cell model.

Our results suggest that the patient-specific genetic background could be a critical determinant of the phenotypical manifestation of Brugada syndrome. Finally, our work highlights the need of assessing the pathophysiological mechanisms of sodium channel mutations in a cardiac- and patient-specific model.

## 5 Study limitations

Our results illustrate the ability of patient-specific iPS cell technology to model the abnormal functional phenotype of Brugada syndrome. Nevertheless, an issue still remains, which is the maturity of the obtained iPS-CM. Although cardiomyocytes derived from iPS cells usually represent a mixture of cells with different degrees of maturity, it has been demonstrated that human iPS-CM have all major cardiac ionic currents, including *I*_Na_, *I*_Ca-L_, *I*_f_, *I*_to_, *I*_K1_, *I*_Kr_, *I*_Ks_ and *I*_K,ATP_ [28], [36], [37] and would thus be suitable for examining ion channel function in a patient-specific context. In this sense, we have shown that the cardiomyocytes obtained from our patient and control exhibit typical cardiac markers and have functional cardiac sodium channels. Therefore, our results support the accepted current idea that the relative immaturity of iPS-CM does not prevent their use as an accurate system to model channelopathies affecting Na_v_1.5. Nevertheless, due to this limitation, we focused our studies on the sodium current since this is less subject to cell variability than action potentials.

It is important to acknowledge that we cannot generalize our results to other sodium channel mutations. Thus, at the moment our conclusions should be limited to the specific variant studied in this work. Nonetheless, we believe that our findings represent important evidence on how the cellular environment may contribute to the phenotypical expression of a sodium channel mutation.

## 6 Conclusions

Cardiomyocytes derived from iPS cells from a Brugada syndrome patient with a mutation in *SCN5A* recapitulate the loss of function of the sodium channel current associated with this syndrome, including pro-arrhythmic changes in channel function that could only be detected using iPS-CM and not in conventional heterologous expression systems. We believe that our findings underscore the extra value that the use of patient-specific iPS-CM confers to mutation studies in BrS. Therefore, we expect that our work will contribute to establish iPS-CM as an adequate cell model for the investigation of BrS.

The following are the supplementary data related to this article.

Supplementary Video 1 Video recording of a representative single beating body derived from Control iPS cell line - 2. Supplementary Video 1

Supplementary Video 2 Video recording of a representative single beating body derived from Brugada syndrome iPS cell line - 1. Supplementary Video 2

Supplementary Video 3 Video recording of a representative beating sheet derived from Control iPS cell line - 7. Supplementary Video 3

Supplementary Video 4 Video recording of a representative beating sheet derived from Brugada syndrome iPS cell line - 1. Supplementary Video 4

Supplementary material Image 1

## Disclosures

None.
