# Supplementary material for: Sodium channel current loss of function in induced pluripotent stem cell-derived cardiomyocytes from a Brugada syndrome patient
Source: J Mol Cell Cardiol. 2018 Jan;114:10–9. doi: 10.1016/j.yjmcc.2017.10.002 (PMC5807028; doi:10.1016/j.yjmcc.2017.10.002)
Supplement: Supplementary file 5 — Supplementary material [file mmc5.pdf]

Supplemental Material

Sendfeld *et al.*, Sodium Current Loss of Function in Induced Pluripotent Stem Cell-derived Cardiomyocytes from a Brugada Syndrome Patient.

1. Detailed Materials and Methods

1.1 Skin biopsy processing and isolation of fibroblasts

This study was approved by the South East Scotland Research Ethics Committee (REC reference 11-SS-0095) and written informed consent was obtained from all subjects. Dermal biopsies were collected in 10% FCS medium (Dulbecco's Modified Eagle's Medium (DMEM) supplemented with 10% FCS, Pen-Strep (50 U/mL Penicillin, 50 µg/mL Streptomycin) and 2mmol/L L-Glutamine, all from Life Technologies- Thermo Fisher Scientific Inc., Waltham, MA, USA) on ice and processed within an hour of collection. Tissue was transferred into minimal volume of fibroblast growth medium before dissecting off the epidermis and fat with sterile scalpels. The remaining tissue was dissected into 1 mm^2 pieces. Four tissue pieces were transferred to each well of a tissue culture treated 6-well plate, covered by a sterile coverslip, leaving room for cell expansion, and slowly submerged in fibroblast growth medium (DMEM plus GlutaMax supplemented with 10% HyClone FBS (GE Healthcare Life Sciences, Utah, USA), PenStrep, 50 µg/mL Gentamicin and 1.25 µg/mL Amphotericin B, all from Life Technologies). The tissue pieces were cultured at 37°C with 5% CO_2 with the first medium change carried out after 7 days and subsequently every 4-5 days. Once fibroblasts had grown out from the tissue and expanded, they were harvested using 0.25% Trypsin/EDTA, pelleted at 1000 rpm for 5 min, resuspended in medium and cultured in tissue culture treated flasks with media changes every other day.

1.2 Reprogramming of fibroblasts to induced pluripotent stem (iPS) cells

Fibroblasts were expanded, harvested as described above and counted using a haemocytometer. 5 x 10^5 cells were electroporated in an Amaxa Nucleofector 2b (Lonza, Basel, Switzerland) using the Amaxa NHDF Nucleofector Kit (Lonza) and 5µg DNA (1.7 µg pCXLE-hOCT3/4-shp53-F (Addgene plasmid # 27077), 1.6 µg pCXLE-hSK (Addgene plasmid # 27078) and 1.7 µg pCXLE-hUL (Addgene plasmid # 27080); all plasmids were a gift from Shinya Yamanaka[1], following the manufacturer's recommendations, and plated in one well of a 6-well plate. After 24 hours and then again 4 days post electroporation, medium was replaced with 10% FCS medium. Seven days after electroporation cells were lifted with Trypsin/EDTA and re-plated into a 0.1% gelatin coated tissue culture treated 10 cm culture dish in 10% FCS medium. After 24 hours, medium was replaced with stem cell selection medium TeSR-E8 (STEMCELL Technologies SARL, Grenoble, France). Media changes were subsequently carried out every two days and cell morphology was assessed using a Nikon Eclipse TE300 light microscope. Genuine iPS cell colonies appeared between 20-25 days

after electroporation and were manually picked into separate wells of Matrigel (BD Biosciences, Franklin Lakes, NJ, USA) coated 4 well plates in TeSR-E8 medium. Colonies were treated as individual clones and expanded to give rise to independent cell lines.

1.3 iPS cell maintenance

Cells were maintained on Matrigel coated plates in TeSR-E8 medium at 37°C and 5% CO_2. Media changes were carried out daily and cells were passaged chemically and mechanically using sterile cell scrapers and 0.5 mmol/L EDTA (LifeTechnologies).

1.4 Sequencing

The patient chosen for this study had previously undergone genetic testing which identified the *SCN5A*_c.1100G>A variation as the likely cause for his condition. To confirm the presence of this variation in the iPS cell lines derived from the patient and its absence from the iPS cell lines derived from the healthy volunteers, the whole coding region of *SCN5A* was amplified (Verities PCR, Applied Biosystems, Austin, TX, USA), the PCR products were purified (ExoSAP-IT, Affymetrix, Inc. USB® Products, Cleveland, OH, USA) and they were directly sequenced in both directions (Big Dye Terminator v3.1 cycle sequencing kit and 3130XL Genetic Analyzer, both from Applied Biosystems). DNA sequences obtained were compared with *SCN5A* reference sequence NM_198056.2 using SeqScape v2.6 (Applied Biosystems).

1.5 Real-time quantitative reverse transcription PCR (qPCR)

RNA extraction and cDNA synthesis were carried out using the MasterPure™ Complete DNA & RNA Purification Kit (Cambio, Cambridge, UK) and High Capacity cDNA Reverse Transcription Kit (Applied Biosystems-Thermo Fisher Scientific) according to manufacturer's specifications.

qPCR reactions were set up using the GoTaq qPCR Master Mix kit (Promega, Madison, WI, USA) per the manufacturer's instructions, and including a reference dye. The samples were analyzed in biological triplicates using the primers listed in Supplemental Table 2 and run in a Rotor-Gene 6000 series software 1.7 (Corbett Life Science-Qiagen, Manchester, UK).

All fluorescence signals were normalized to reference dye fluorescence signals before further analysis. Cycle threshold (Ct) values were averaged for the technical triplicates, and expression levels of the gene of interest, normalized to β-actin, were determined by the ΔΔCt method. Biological triplicates were averaged, standard errors of the mean were calculated and graphs were plotted using GraphPad Prism 5.00. The relative expression of the gene of interest in different cell lines was compared using a one-way ANOVA with either the Dunnett's or Tukey post-test.

1.6 Flow cytometry

Pluripotent stem cells at ~80% confluency were lifted with TrypLE Select (LifeTechnologies), counted and double stained using antibodies against SSEA-3 (Alexa Fluor 488 conjugate,

from BioLegend, San Diego, CA, USA) and SSEA-4 (Phycoerythrin conjugate, from BD Biosciences), 1:50 dilution for 30min at room temperature in the dark. Cells were then washed with PBS and resuspended in 10% BD FACS Lysing solution (BD Biosciences) and stored at 4°C for up to 7 days before flow cytometry analysis.

Samples were run on a FACS Fortessa Flow Cytometry System acquiring a minimum of 10,000 events. Flow cytometry data was analyzed using FlowJo (Treestar, Inc., San Carlos, CA, USA). Color compensation was applied to compensate for overlapping emission spectra of Phycoerythrin and Alexa Fluor 488. Results from three independent replicates for each cell line were pooled, expressed as the mean with standard error of the mean (SEM) and plotted.

1.7 Immunostaining for pluripotency markers

Undifferentiated cells were fixed in 4% paraformaldehyde (PFA), permeabilized with 0.1% Triton X-100 (Sigma-Aldrich, St. Louis, MO, USA) in PBS for 10 min, blocked with 3% horse serum in PBS for 30 min and stained for 1 hour with antibodies against Nanog (R&D Systems, Abingdon, UK, 1:100), Tra-1-60 or Oct-4 (both from SantaCruz Biotechnology, Dallas, TX, USA, 1:200 and 1:400, respectively) diluted in 3% horse serum in PBS. Samples were then incubated for 1 hour with Rabbit anti goat Alexa Fluor 568 (Nanog) or Goat anti mouse Alexa Fluor 488 (Tra-1-60 and Oct-4), all of them 1:400 in PBS, and counterstained and mounted with ProLong Gold Antifade Reagent with DAPI.

1.8 Single Nucleotide Polymorphism (SNP) analysis

Confluent wells of undifferentiated pluripotent stem cells were harvested with EDTA and pelleted at 200g for 5min. Cells were washed with PBS, centrifuged again and dry pellets were stored at -20°C. Genomic DNA was isolated from cell pellets using the MasterPure™ Complete DNA & RNA Purification Kit (Cambio) according to manufacturer's specifications. Purified genomic DNA (500ng) was sent to AROS Applied Biotechnology A/S (Aarhus, Denmark) for single nucleotide polymorphism (SNP) analysis. The employed SNP array, Illumina CytoSNP-12, incorporates 294,975 markers.

The SNP array data was visualized and analyzed using the Illumina Genome Viewer 1.9.0 of the Illumina GenomeStudio V2011.1 software. The Log R ratio and β allele frequency (BAF) were plotted and analyzed for presence of microduplications and deletions. The Log R ratio was calculated as the log2 (sample/reference). The BAF was calculated as (B / (A + B)) with A and B representing the signals for each SNP. Each SNP was plotted according to its genotype Log R ratio and BAF were analyzed for each chromosome for each cell line.

1.9 Three germ layer differentiation and staining

Embryoid bodies (EBs) were formed from confluent undifferentiated pluripotent stem cell colonies. After a 30 s incubation with Collagenase IV at 37°C, colonies were washed with PBS and mechanically disrupted with a sterile pipette to yield evenly sized clumps. These clumps were harvested, transferred onto non-treated tissue culture dishes and cultured in suspension in EB medium (DMEM supplemented with 10% FCS, PenStrep, NEAA, 2 mmol/L L-Glutamine

and 0.1 mmol/L β-Mercaptoethanol) at 37°C and 5% CO_2. Medium was changed every other day by letting EBs settle in a conical tube, carefully aspirating supernatant and resuspending in new medium. On day 7, EBs were plated onto 0.1% gelatin coated tissue culture treated plates and cultured for a further 10 days, continuing media changes every other day. EBs were fixed in ice-cold methanol, permeabilized and blocked with 10% goat serum in PBST (PBS supplemented with 0.1% Tween 20) for 45 min, washed twice with PBS and incubated for 1h with antibodies against α-fetoprotein, β-tubulin III (both from Sigma-Aldrich, 1:500 and 1:1000, respectively) or Muscle actin (Dako, Glostrup, Denmark, 1:50) diluted in 1% goat serum in PBST. After 2 PBS washes, samples were incubated with Goat anti mouse Alexa Fluor 488 1:400 in PBS for 1 hour, and counterstained and mounted with ProLong Gold Antifade Reagent with DAPI (Life Technologies).

1.10 Cardiac differentiation

EBs were formed in differentiation medium (KO-DMEM supplemented with 20% HyClone FCS, PenStrep, NEAA, 2 mmol/L L-Glutamine, 0.1 mmol/L β-Mercaptoethanol, 5 µmol/L SB203580 (Tocris, 1202) and 2 µmol/L PGI2 (Sigma-Aldrich)) using the technique described above and cultured in suspension for 4 days before being plated onto 0.1% gelatin coated tissue culture treated 6 well plates. Differentiation medium was changed every 3 days. Beating bodies started to emerge around day 8 after EB formation.

We also used a monolayer-based protocol [2]. iPS cells were allowed to grow to 85-90% confluence. Then, culture media was changed to differentiation medium (DMEM/F12 supplemented with 64mg/L L-Ascorbic acid, 14µg/L sodium selenite, 10µg/ml Holo-Transferrin, Chemically defined Lipid concentrate, Glutamax and PenStrep) containing 4-6µmol/L CHIR99021 (Day 0). Twenty-four hours later, media was changed to differentiation medium with 3µg/ml heparin. From day 2 to day 5 the media contained 3µg/ml heparin and 3µmol/L IWP2, and was renewed daily. At day 6, media was changed to differentiation medium with 3µg/ml heparin only. The day after, the differentiation medium contained 3µg/ml heparin and 10µg/ml insulin. From day 8 onwards, cells were fed differentiation medium with 10µg/ml insulin, and media was renewed every 2 days. iPS-CM started beating around day 8-10.

1.11 Beating body and monolayer iPS-CM disaggregation

Buffers for beating body disaggregation were prepared based on the methods published by Maltsev et al.,[3] and Mummery et al.,[4] as listed in Supplemental Table 3, filter-sterilized and stored at -20°C in 1mL aliquots. Glucose was added to each aliquot after thawing. Beating areas were dissected, transferred into Buffer 1 and incubated at room temperature for 30 min. Bodies were then incubated in Buffer 2 at 37°C for 25-45 min, depending on their size, before being transferred to Buffer 3 and incubated at 37°C for 1 hour. Finally, beating bodies were transferred to 10% FCS medium (DMEM supplemented with 10% FCS, PenStrep and 2 mmol/L L-Glutamine), carefully broken up by pipetting 8-12 times using a P1000 pipette and plated onto 1% gelatin. After 24 hours, the medium was topped up and cardiomyocytes started to spontaneously contract 24-48 hours post disaggregation.

iPS-CM monolayers were incubated with 0.25% trypsin-EDTA at 37ºC for 5-6 minutes. Digestion was stopped by addition of differentiation medium with insulin and 20% FBS. The

monolayer was broken into single cells by pipetting using a P1000 pipette. Single iPS-CM were centrifuged at 1000 rpm for 5 min, cell pellet was resuspended in differentiation medium with insulin, 20% FBS and 10nmol/L Y27632, and cardiomyocytes were plated onto 0.1% gelatin. After 24 hours, the medium was replaced by differentiation medium with insulin.

1.12 Immunostaining for cardiac markers

Disaggregated beating bodies were fixed in 4% PFA, permeabilized in 0.5% Triton X-100 in PBS for 10 min, and blocked with 3% goat serum for 30 min. Samples were incubated with antibodies against Cardiac troponin T, Cardiac troponin I (both at 1:100) or Alpha actinin (1:300, all from Abcam, Cambridge, UK). Samples were then incubated for 1 hour with Goat anti mouse Alexa Fluor 488 (Cardiac troponin T and Alpha actinin) or Goat anti rabbit Alexa Fluor 568 (Cardiac troponin I) 1:400 in PBS and counterstained and mounted with ProLong Gold Antifade Reagent with DAPI. Cells grown on plastic were subsequently covered with coverslips. If cells had been grown on coverslips, these were transferred onto glass slides, cells facing down.

1.13 Transmission electron microscopy (TEM)

Beating bodies were fixed with 2.5% Glutaraldehyde (Sigma-Aldrich) in 4% PFA for 1hour at room temperature, then washed for 3 x 15 min in 0.1 mol/L Phosphate buffer (10.22 g Sodium phosphate dibasic anhydrous, 3.9 g Sodium phosphate monobasic dehydrate in 1 L of MiliQ water, pH 7.2). Samples were then post-fixed in 1% Osmium Tetroxide in 0.1mol/L Phosphate buffer for 1 hour, washed three times in 0.1mol/L Phosphate buffer and dehydrated in 50%, 70% and 90% ethanol. Dehydration was finalized with two washes in 100% ethanol and one wash in propylene oxide (Sigma-Aldrich). The tissue was transitioned to embedding resin in a 1:1 (v/v) mixture of propylene oxide and overnight resin (1:1 (w/w) Araldite CY212 and Dodecenyl Succinic Anhydride (DDSA)) for 1hour at room temperature on a carousel. After propylene oxide had evaporated, the tissue was transferred to overnight resin and left overnight on a carousel. The next day, the overnight resin was changed every 1.5 hours for 4.5 hours after which it was replaced with final embedding resin (11.5 g Araldite CY212, 11 g DDSA, 0.55 mLs Benzyl Dimethylamine (BDMA), 0.5 mL Dibutylphthalate). Final embedding resin was changed every hour for 3 hours to ensure complete replacement of the previous resin mixture. For the final embedding step tissues were transferred to fresh final embedding resin and left in a 60°C oven for 48 hours in order for the resin to fully harden for sectioning. Sections, 1µm thick, were cut on a Reichert OMU4 ultramicrotome, stained with Toluidine Blue, and viewed in a light microscope to select suitable areas for investigation. Ultrathin sections, 60nm thick, were cut from selected areas, stained in Uranyl Acetate and Lead Citrate then viewed in a Philips CM120 Transmission electron microscope. Images were taken on a Gatan Orius CCD camera.

1.14 Sodium current recordings in iPS-CM

Beating bodies were disaggregated and plated onto gelatin as described above. Electrophysiological recordings were performed on single cells, identified as cardiomyocytes by their morphology and/or spontaneous contraction, 24-48h after disaggregation. Whole cell

sodium currents were measured at room temperature using the perforated patch-clamp technique. The bath solution contained (mmol/L): 40 NaCl, 100 N-Methyl-D-glucamine, 3 KCl, 10 N-2-hydroxyethylpiperazine- N' -2-ethanesulfonic acid (HEPES), 1.8 CaCl2 and 1.2 MgCl2 (pH 7.4, HCl), and the pipette solution (mmol/L): 130 Aspartic acid, 10 NaCl, 10 HEPES, 1 Ethylene glycol-bis(2-amino-ethylether)-N,N, N',N'-tetra-acetic acid (EGTA), 2 MgCl2 (pH 7.2, CsOH) and 240μg/ml Amphotericin B. Osmolality was adjusted by the addition of glucose to 323 and 305 mOsm for bath and pipette solution, respectively. Pipettes were pulled from glass capillaries (Sutter Instruments Co, CA, USA) and their resistance ranged from 2.8 to 3.5 MΩ when filled with the internal solution.

Beating monolayers were disaggregated and plated onto gelatin as described above. Sodium currents were measured at room temperature using the standard whole cell patch-clamp technique [5] 24-96h after disaggregation. The bath solution contained (mmol/L): 140 NaCl, 3 KCl, 10 N-2-hydroxyethylpiperazine- N' -2-ethanesulfonic acid (HEPES), 1.8 CaCl2 and 1.2 MgCl2 (pH 7.4, NaOH); and the pipette solution (mmol/L): 130 CsCl, 1 Ethylene glycol-bis(2-amino-ethylether)-N,N, N',N'-tetra-acetic acid (EGTA), 10 HEPES, 10 NaCl and 2 ATP Mg2+(pH 7.2, CsOH). Osmolality was adjusted by the addition of glucose to 325 and 308 mOsm for bath and pipette solution, respectively. Pipettes were pulled from glass capillaries (Sutter Instruments Co) and their resistance ranged from 2.5 to 3.2 MΩ when filled with the internal solution. Series resistance was compensated 80–90%.

Voltage clamp experiments were controlled and analyzed with an Axopatch 200B amplifier and pClamp 10.2/Digidata 1440A acquisition system (Molecular Devices, Sunnyvale, CA, USA) and OriginPro8 software (OriginLab Corporation, Northampton, MA, USA). Data were filtered at 5 kHz and sampled at 5–20 kHz. Activation curve data were fitted to a Boltzmann equation, of the form $g=g_{max}/(1+ \exp(V_{1/2}-V_m)/k)$, where g is the conductance, g_{max} the maximum conductance, V_m is the membrane potential, $V_{1/2}$ is the voltage at which half of the channels are activated and k is the slope factor. Steady-state inactivation values were fitted to a Boltzmann equation of the form $I=I_{max}/(1+ \exp(V_{1/2}-V_m)/k)$, where I is the peak current amplitude, I_{max} the maximum peak current amplitude, V_m is the membrane potential, $V_{1/2}$ is the voltage at which half of the channels are inactivated, and k is the slope factor. The sodium current decay after the peak I_{Na} was fitted with a monoexponential function between −40 and −25 mV, and a bi-exponential function between −20 and 20 mV, from where τ fast and τ slow were obtained. Both the slow inactivation and the recovery from inactivation data were fitted to mono-exponential functions, to obtain their respective time constants.

1.15 Sodium current recordings in tsA201 cells

1.15.1 Site-directed mutagenesis

SCN5A_c.1100G>A was introduced in the wild-type (WT) human *SCN5A* cDNA (Uniprot reference: Q14524) cloned in pcDNA3.1 (a kind gift from Dr. Matteo Vatta, Baylor College of Medicine, Houston, TX, USA) using the QuikChange Site-Directed Mutagenesis system (Stratagene, La Jolla, CA, USA) and the following primers (mutation underlined):
5'- GCCTTTCTTGCACTCTTCCACCTGATGACGCAGGACTGC -3'
5'- GCAGTCCTGCGTCATCAGGTGGAAGAGTGCAAGAAAGGC -3'

The resultant construct was directly sequenced to verify the presence of the desired mutation and the absence of additional variations.

1.15.2 Cell culture and transfection

tsA201 cells were maintained in Dulbecco's Modified Eagle's Medium supplemented with 10% Fetal Bovine Serum, 1% antibiotic-antimycotic and 1% Glutamax (all from Invitrogen, Carlsbad, CA, USA) at 37ºC and 5% CO_2. Cells were transiently transfected with the vectors encoding for *SCN5A* (WT only, R367H only, or both WT and R367H vectors), using Lipofectamine 2000 (Life Technologies) following the manufacturer's specifications. Co-transfection with a plasmid containing the green fluorescent protein (GFP) gene (a kind gift from Dr. Kirstine Calløe, University of Copenhagen, Copenhagen, Denmark) allowed the identification of transfected cells.

1.15.3 Electrophysiological studies

Sodium currents were measured at room temperature using the standard whole cell patch-clamp technique [5] 48 hours after transfection. The bath solution contained (mmol/L): 140 NaCl, 3 KCl, 10 N-2-hydroxyethylpiperazine- N' -2-ethanesulfonic acid (HEPES), 1.8 CaCl2 and 1.2 MgCl2 (pH 7.4, NaOH); and the pipette solution (mmol/L): 130 CsCl, 1 Ethylene glycol-bis(2-amino-ethylether)-N,N, N',N'-tetra-acetic acid (EGTA), 10 HEPES, 10 NaCl and 2 ATP Mg2+(pH 7.2, CsOH). Osmolality was adjusted by the addition of glucose to 325 and 308 mOsm for bath and pipette solution, respectively. Pipettes were pulled from glass capillaries (Brand GMBH+CO KG, Wertheim, Germany) and their resistance ranged from 2.5 to 3.2 MΩ when filled with the internal solution. Series resistance was compensated 80–90%. Voltage clamp experiments were controlled and analyzed as described for iPS-derived cardiomyocytes.

1.16 Cell Surface Protein Biotinylation in tsA201 cells

Cells were washed with Dulbecco's Phosphate-Buffered Saline (DPBS) supplemented with 0.9 mmol/L CaCl2 and 0.49 mmol/L MgCl2 (DPBS+) at pH 7.4. Membrane proteins were biotinylated by incubating cells with 1.6–2.5 mg/ml of EZ-link sulfo-NHS-LCLC- biotin (Pierce, Thermo Scientific, Rockford, IL, USA) in DPBS+ for 30 min at 4ºC. Cells were then washed 3 times in DPBS+ with 100 mmol/L glycine, then with DPBS+ containing 20 mmol/L glycine, and scrapped in Triton X-100 lysis buffer (1% Triton X-100, 50 mmol/L Tris/HCl pH 7.4, 150 mmol/L NaCl, 1 mmol/L EDTA and Complete Protease Inhibitor Cocktail (Roche, Madrid, Spain)). Lysates were obtained after 1 h rotating at 4ºC. Insoluble materials were removed by centrifugation. Supernatants were incubated with Ultralink Immobilised NeutrAvidin beads (Pierce) overnight at 4ºC. The beads were precipitated and washed with Triton X-100 lysis buffer, then in saline solution (5 mmol/L EDTA, 350 mmol/L NaCl and 0.1% TX-100 in DPBS+ pH 7.4) and finally in 10 mmol/L Tris/HCl pH 7.4. Precipitated beads were resuspended in SDS-PAGE loading buffer and heated for 5 min at 70ºC. Proteins were resolved in 4% SDS-PAGE gels and transferred to PVDF membranes (Millipore, Billerica, MA, USA). Membranes were probed with a rabbit anti-human Nav1.5 antibody (anti-hNav1.5; Alomone Labs, Jerusalem, Israel) at a dilution of 1:1,000, overnight at 4ºC. A secondary horseradish peroxidase-conjugated antibody (Thermo Scientific, Rockford, IL, USA) was used at a dilution

of 1:2,000 for 1 h at room temperature, and signals were detected with the SuperSignal West Femto Chemiluminiscent substrate (Pierce). A mouse antibody against Na+/K+ ATPase (Abcam) was used as biotinylation control. Protein markers for molecular weights from 10 to 250 kDa (PageRuler^{TM} Plus Prestained Protein Ladder, Thermo Scientific) were used as size standards in protein electrophoresis (SDS-PAGE) and Western blotting. Expression of Nav1.5 was quantified using the ImageJ software (National Institute of Health, NIH) available at http://rsb.info.nih.gov/ij. Intensity values for each band were determined as the integrated density (sum of pixel values) within a fixed area. To account for differences due to loading, Nav1.5 intensity values were normalized with Na+/K+ ATPase intensity values. Then, all normalized values were divided by the mean of normalized intensity values for the WT/WT cells.

1.17 Statistical Analysis

Results are presented as means ± standard error. Statistical comparisons were performed using an unpaired Student's t-test or two-way ANOVA with a Bonferroni or Dunnett's post-test for multiple comparisons as appropriate. Statistical significance was defined where $p<0.05$.

2. Supplemental References

[1] Okita K, Matsumura Y, Sato Y, Okada A, Morizane A, Okamoto S, et al. A more efficient method to generate integration-free human iPS cells. Nat Methods. 2011;8:409-12.
[2] Lin Y, Linask KL, Mallon B, Johnson K, Klein M, Beers J, et al. Heparin Promotes Cardiac Differentiation of Human Pluripotent Stem Cells in Chemically Defined Albumin-Free Medium, Enabling Consistent Manufacture of Cardiomyocytes. Stem Cells Transl Med. 2017;6:527-38.
[3] Maltsev VA, Wobus AM, Rohwedel J, Bader M, Hescheler J. Cardiomyocytes differentiated in vitro from embryonic stem cells developmentally express cardiac-specific genes and ionic currents. Circ Res. 1994;75:233-44.
[4] Mummery CL, Ward D, Passier R. Differentiation of human embryonic stem cells to cardiomyocytes by coculture with endoderm in serum-free medium. Curr Protoc Stem Cell Biol. 2007;Chapter 1:Unit 1F.2.
[5] Hamill OP, Marty A, Neher E, Sakmann B, Sigworth FJ. Improved patch-clamp techniques for high-resolution current recording from cells and cell-free membrane patches. Pflugers Arch. 1981;391:85-100.

3. Supplemental Tables

Supplemental Table 1: Chromosomal abnormalities identified through SNP analysis and the genes affected

Cell line	Region affected	Genes in the region affected
Control iPS cells - 1	7q11.21 (62.139.338 - 62.834.662)	None
	14q23.2 (63.569.297 - 63.668.333)	None
Control iPS cells - 2	20q11.21	ID1 (inhibitor of DNA binding 1)<br>BCL2L1 (Bcl-2-like protein 1)<br>HM13 (Minor histocompatibility antigen H13)
Control iPS cells - 3	20q11.21	ID1 (inhibitor of DNA binding 1)<br>BCL2L1 (Bcl-2-like protein 1)<br>HM13 (Minor histocompatibility antigen H13)
Control iPS cells - 4	None	None
Control iPS cells - 5	None	None
Control iPS cells - 6	None	None
Brugada syndrome iPS cells - 1	None	None
Brugada syndrome iPS cells - 2	5q33.2 (155.453.475 - 155.967.030)	SGCG (delta-sarcoglycan)
Brugada syndrome iPS cells - 3	2q31.1 (171.032.090 - 171.435.002)	MYO3B (myosin IIIB)
	3p13 (71.688.564 - 74.289.885)	PROK2 (prokineticin 2 isoform a precursor)<br>GPR27 (G protein coupled receptor 27)<br>EIF4E3 (eukaryotic translation initiation factor 4E)<br>RYBP (RING1 and YY1 binding protein)<br>SHQ1 (SHQ1 homolog)<br>GLT8D4 (glycosyltransferase 8 domain containing 4)<br>PPP4R2 (protein phosphatase 4, regulatory subunit 2)<br>FLJ10213 (hypothetical protein LOC55096)<br>PDZRN3 (PDZ domain containing ring finger 3)
	4q32.1 (155.654.421 - 155.938.012)	LRAT (lecithin retinol acyltransferase),<br>RBM46 (RNA binding motif protein 46)<br>Putative uncharacterized protein MGC27016

	12p13.1 (13.394.582 - 13.446.880)	None
	17q12 (36.821.619 - 37.024.319)	C17orf96 (hypothetical protein LOC100170841)<br>MLLT6 (myeloid/lymphoid or mixed lineage leukemia)<br>CISD3 (CDGSH iron sulfur domain 3 precursor)<br>PCGF2 (ring finger protein 110)<br>PSMB3 (proteasome beta 3 subunit)<br>PIP4K2B (phosphatidylinositol 5 phosphate 4 kinase, type)<br>CCDC49 (coiled coil domain containing 49)<br>C17orf96 (hypothetical protein LOC388381)<br>RPL23 (ribosomal protein L23)<br>SNORA21 (Homo sapiens small nucleolar RNA, H/ACA box 21 (SNORA21), non coding RNA.)
Brugada syndrome iPS cells - 4	None	None
Brugada syndrome iPS cells - 5	None	None

The table shows the chromosomal abnormalities that were identified in the control and Brugada syndrome iPS cell lines. All the alterations identified were microduplications. The chromosome, band and precise position of the abnormalities found are provided. Also, the genes in the region affected and their description are also supplied.

Supplemental Table 2: Primers used for qPCR

Gene	Sequence forward and reverse primers (5'->3')	Final concentration
β-actin	CAAACATGATCTGGGTCATCTT<br>GCTCGTCGTCGACAACGGCTC	100 nmol/L
c-Myc	TACCCTCTCAACGACAGCAG<br>GTCTCCTCATGGAGCACCAGG	250 nmol/L
Nanog	GACAAGGTCCCGGTCAAGAA<br>CTGAGGTTCAGGATGTTGGA	250 nmol/L
Oct-4	CCCGCCGTATGAGTTCTGTG<br>CATCGGAGTTGCTCTCCACC	50 nmol/L
Sox2	AGTCCGAGGCCAGCTCCA<br>TAGTGCTGGGACATGTGAAGTC	250 nmol/L

The table provides the sequences for the amplification of the pluripotency genes using qPCR, and the final concentrations that they were used.

Supplemental Table 3: Composition of buffers used to disaggregate beating bodies

Component	Buffer 1	Buffer 2	Buffer 3
NaCl	120 mmol/L	120 mmol/L	-
KCl	5.4 mmol/L	5.4 mmol/L	85 mmol/L
$MgSO_4$	5 mmol/L	5 mmol/L	5 mmol/L
Na pyruvate	5 mmol/L	5 mmol/L	5 mmol/L
Taurine	20 mmol/L	20 mmol/L	20 mmol/L
HEPES	10 mmol/L	10 mmol/L	-
$CaCl_2$	-	30μmol/L	-
Collagenase B	-	1 mg/mL	-
EGTA	-	-	1 mmol/L
Creatine	-	-	5 mmol/L
K_2HPO_4	-	-	30 mmol/L
Na_2ATP	-	-	2 mmol/L
pH (NaOH)	6.9	6.9	7.2

The composition of the 3 buffers used to disaggregate the beating bodies is provided. Reagents were obtained from Sigma-Aldrich, except for Collagenase B, which was purchased from Roche (West Sussex, UK). Glucose (20 mmol/L) was added to the 3 buffers right before use.

4. Supplemental Video captions

Supplemental Video 1. Video recording of a representative single beating body derived from Control iPS cell line - 2.

Supplemental Video 2. Video recording of a representative single beating body derived from Brugada syndrome iPS cell line - 1.

Supplemental Video 3. Video recording of a representative beating sheet derived from Control iPS cell line - 7.

Supplemental Video 4. Video recording of a representative beating sheet derived from Brugada syndrome iPS cell line - 1.

5. Supplemental Figures

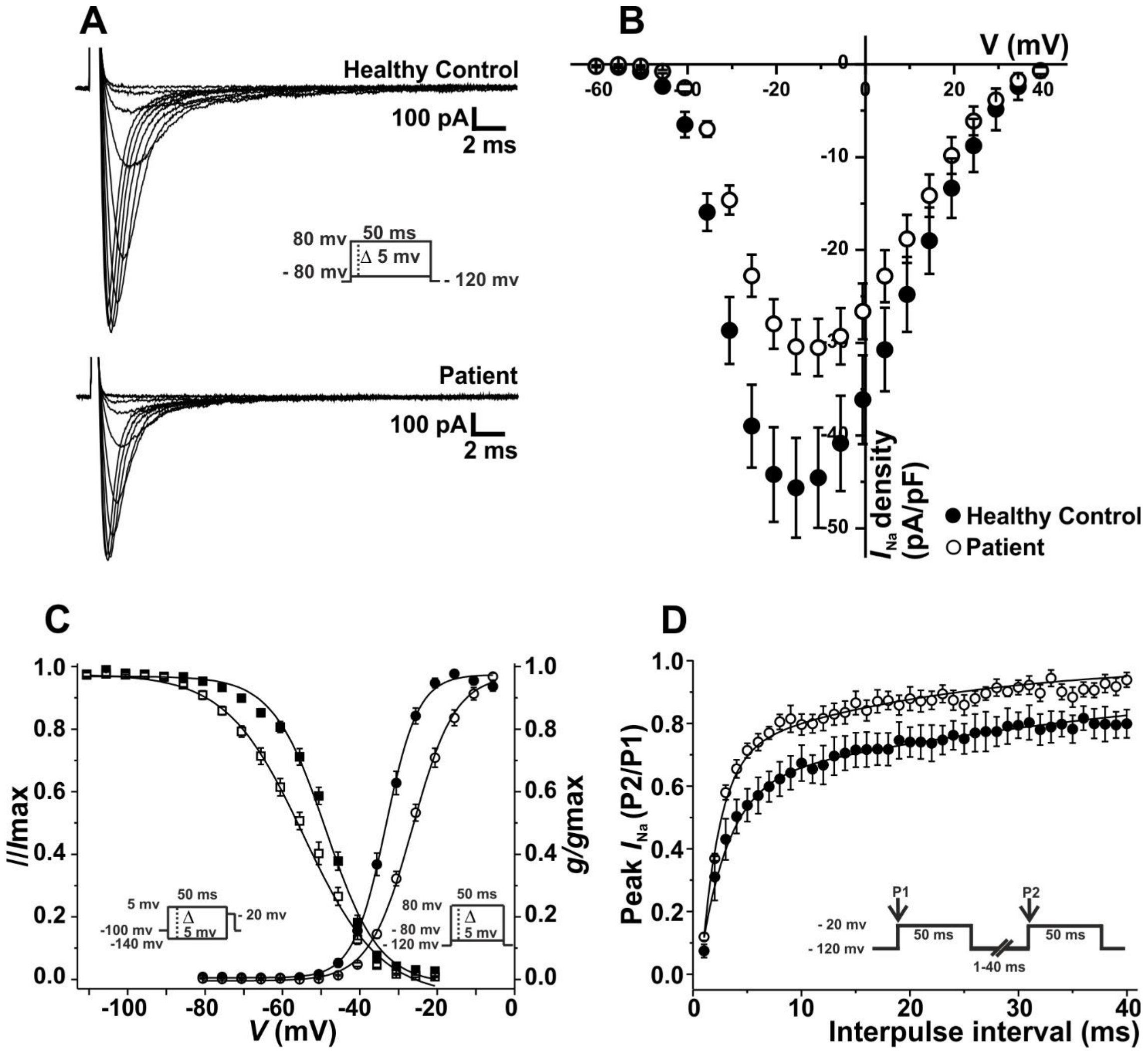

Supplemental Fig 1. Monolayer-derived patient-specific iPS-CM display altered I_{Na} properties. Filled symbols are used to depict data for healthy control iPS-CM and open symbols represent values for patient-specific cardiomyocytes. Values are expressed as mean ± SEM. **(A)** Representative whole cell sodium current traces recorded from control and patient-specific cells. Currents were elicited by depolarizing potentials as shown in the inset. Traces for pulses from -45 mV to -5 mV are shown. **(B)** Current-voltage (*I–V*) relationship. I_{Na} amplitude was normalized to the cell capacitance to obtain current density (I_{Na} density) values. Experimental points represent the peak-amplitude of I_{Na} density at each given voltage. **(C)** I_{Na} voltage-dependence of activation and steady-state inactivation for control and patient cells. Conductance values for the activation curve were obtained from the peak current values taken from the *I–V* relationship. Symbols represent experimental data plotted against the given depolarizing voltage values. Steady-state inactivation protocol is shown in the inset on the left. Symbols represent experimental data plotted against preconditioning pulse values. Solid lines represent the Boltzmann fit of the experimental points. **(D)** Recovery from inactivation properties were studied by applying the double pulse protocol shown in the inset. A 50 ms depolarizing pulse to -20 mV (P1) was followed by a hyperpolarizing pulse to -120 mV of increasing duration (1–40 ms), that preceded a test pulse to -20 mV (P2). The P2/ P1 ratio values plotted against the recovery interval times were fitted to bi-exponential functions (solid lines).
